# The Geometry of *m*-Hyperconvex Domains

**DOI:** 10.1007/s12220-017-9957-2

**Published:** 2017-11-16

**Authors:** Per Åhag, Rafał Czyż, Lisa Hed

**Affiliations:** 1 Department of Mathematics and Mathematical Statistics, Umeå University, 901 87 Umeå, Sweden; 2 Faculty of Mathematics and Computer Science, Jagiellonian University, Łojasiewicza 6, 30-348 Kraków, Poland

**Keywords:** Barrier function, Caffarelli–Nirenberg–Spruck model, Exhaustion function, *m*-subharmonic function, Jensen measure, Primary 31C45, 32F17, 32U05, Secondary 31B25, 32U10, 32T35, 46J10, 46A20

## Abstract

We study the geometry of *m*-regular domains within the Caffarelli–Nirenberg–Spruck model in terms of barrier functions, envelopes, exhaustion functions, and Jensen measures. We prove among other things that every *m*-hyperconvex domain admits an exhaustion function that is negative, smooth, strictly *m*-subharmonic, and has bounded *m*-Hessian measure.

## Introduction

The geometry of the underlying space is usually essential when studying a given problem in analysis. The starting point of this paper is the model presented by Caffarelli et al. [16] in 1985 that makes it possible to investigate the transition between potential and pluripotential theories. Their construction relies on Gårding’s research on hyperbolic polynomials [27]. The authors of [16] also provided a very nice application to special Lagrangian geometry, which was in itself introduced as an example within calibrated geometry [30]. With the publications of [9], and [45], many analysts and geometers became interested in the Caffarelli–Nirenberg–Spruck model. To mention some references [23, 37, 47, 49, 51, 64, 71]. A usual assumption in these studies is that the underlying domain should admit a continuous exhaustion function that is *m*-subharmonic in the sense of Caffarelli et al. (see Sect. 2 for the definition of *m*-subharmonic functions). In this paper, we shall study the geometric properties of these domains. Let us now give a thorough background on the motivation behind this paper. It all starts with the following theorem:

### Theorem A

Assume that $\Omega$ is a bounded domain in ${\mathbb {R}}^N$, $N\ge 2$. Then the following assertions are equivalent.

1. $\partial \Omega$ is *regular* at every boundary point $y_0\in \partial \Omega$, in the sense that
  $$\begin{aligned} \lim _{\begin{array}{c} x\rightarrow y_0\\ x\in \Omega \end{array}} {\text {PWB}}_f(x) =f(y_0), \end{aligned}$$
  for each continuous function $f:\partial \Omega \rightarrow {\mathbb {R}}$. Here
  $$\begin{aligned} {\text {PWB}}_f(x)=\sup \Bigg \{v(x): v\in {\mathcal {SH}}(\Omega ),\; \varlimsup _{\begin{array}{c} \zeta \rightarrow \xi \\ \zeta \in \Omega \end{array}}v(\zeta )\le f(\xi ), \;\; \forall \xi \in \partial \Omega \Bigg \}, \end{aligned}$$
  and ${\mathcal {SH}}(\Omega )$ is the space of subharmonic functions defined on $\Omega$;
2. $\partial \Omega$ has a *strong barrier* at every point $y_0\in \partial \Omega$ that is subharmonic, i.e., there exists a subharmonic function $u:\Omega \rightarrow {\mathbb {R}}$ such that
  $$\begin{aligned} \lim _{\begin{array}{c} x\rightarrow y_0\\ x\in \Omega \end{array}} u(x)=0, \end{aligned}$$
  and
  $$\begin{aligned} \limsup _{\begin{array}{c} x\rightarrow y\\ x\in \Omega \end{array}} u(x)<0 \qquad \text { for all } y\in {\bar{\Omega }}\backslash \{y_0\}. \end{aligned}$$
3. $\partial \Omega$ has a *weak barrier* at every point $y_0\in \partial \Omega$ that is subharmonic, i.e., there exists a subharmonic function $u:\Omega \rightarrow {\mathbb {R}}$ such that $u<0$ on $\Omega$ and
  $$\begin{aligned} \lim _{\begin{array}{c} x\rightarrow y_0\\ x\in \Omega \end{array}} u(x)=0. \end{aligned}$$
4. $\Omega$ admits an *exhaustion function* that is negative and subharmonic, i.e., there exists a non-constant function $\psi :\Omega \rightarrow {\mathbb {R}}$ such that for any $c\in {\mathbb {R}}$ the set $\{x\in \Omega :\psi (x)<c\}$ is relatively compact in $\Omega$. Furthermore, the exhaustion function should be negative and subharmonic.
5. $\partial \Omega$ is equal to the *Jensen boundary* w.r.t. the Jensen measures generated by the cone of functions that are continuous on ${{\bar{\Omega }}}$, and subharmonic on $\Omega$ (see Sect. 2 for definitions).

The idea of a regular boundary point can be traced back to 1911 and 1912 with the works of Zaremba [70] and Lebesgue [42], respectively, when they constructed examples that exhibit the existence of irregular points. A decade after these examples, Perron introduced in 1923 the celebrated envelope construction ${\text {PWB}}_{f}$ (see Condition (1)). The work on ${\text {PWB}}_{f}$ was later continued by Wiener [66–68], and in our setting concluded by Brelot [11] in 1939. The notion of barrier goes further back in time; it can be found in the work of Poincaré [53] from 1890. The implication (3) $\Rightarrow$ (1) is due to Bouligand [10] who generalized a result of Lebesgue [43]. The equivalence with assertion (5) originates from the study of function algebras known as Choquet theory, which was developed in the 50’s and 60’s by Bauer, Bishop, Choquet, de Leeuw, and others (see e.g., [25, 28, 29] and the references therein). For a beautiful treatise on Choquet theory we highly recommend [48].

Inspired by the beauty of the equivalences in Theorem A, analysts started to investigate these notions within the model introduced by Lelong [44] and Oka [50] in 1942, where subharmonic functions are changed to plurisubharmonic functions. The unit polydisc in ${\mathbb {C}}^n$, $n\ge 2$, shows that the notions of weak and strong barrier for plurisubharmonic functions are not equivalent. Instead, we have Theorems B and C below, where we assume that $n\ge 2$. If $n=1$, then the two theorems become Theorem A since subharmonic functions are then the same as plurisubharmonic functions.

### Theorem B

Assume that $\Omega$ is a bounded domain in ${\mathbb {C}}^n$, $n\ge 2$. Then the following assertions are equivalent.

1. $\partial \Omega$ is *B-regular* at every boundary point $z_0\in \partial \Omega$, in the sense that
  $$\begin{aligned} \lim _{\begin{array}{c} z\rightarrow z_0\\ z\in \Omega \end{array}} {\text {PB}}_f(z) =f(z_0), \end{aligned}$$
  for each continuous function $f:\partial \Omega \rightarrow {\mathbb {R}}$. Here
  $$\begin{aligned} {\text {PB}}_f(z)=\sup \Bigg \{v(z): v\in \mathcal {PSH}(\Omega ),\; \varlimsup _{\begin{array}{c} \zeta \rightarrow \xi \\ \zeta \in \Omega \end{array}}v(\zeta )\le f(\xi ), \;\; \forall \xi \in \partial \Omega \Bigg \}. \end{aligned}$$
  Here $\mathcal {PSH}(\Omega )$ is the space of plurisubharmonic functions defined on $\Omega$;
2. $\partial \Omega$ has a strong barrier at every point that is plurisubharmonic;
3. $\Omega$ admits an exhaustion function $\varphi$ that is negative, smooth, plurisubharmonic, and such that $\left( \varphi (z)-|z|^2\right)$ is plurisubharmonic.
4. $\partial \Omega$ is equal to the Jensen boundary w.r.t. the Jensen measures generated by the cone of functions that are continuous on ${\bar{\Omega }}$, and plurisubharmonic on $\Omega$.

In 1959, Bremermann [13] adopted the idea from assertion (1) in Theorem A to pluripotential theory (see (1) in Theorem B). He named his construction the Perron–Carathéodory function after the articles [15, 54]. The name did not survive the passage of time, and now it is known as the Perron–Bremermann envelope. Drawing inspiration from Choquet theory, and its representing measures [28, 29, 56], Sibony proved Theorem B in the article [58], which was published in 1987. There he also put these conditions in connection with Catlin’s property (*P*), and the ${\bar{\partial }}$-Neumann problem. The last condition in assertion (3) means that we have that

$$\begin{aligned} \sum _{j,k=1}^{n}\frac{\partial ^2\varphi }{\partial z_j\partial {\bar{z}}_k} \alpha _j{\bar{\alpha }}_k\ge |\alpha |^2\,, \;\text { for all }\alpha \in {\mathbb {C}}^n. \end{aligned}$$

Hence, one can interpret $\varphi$ as being *uniformly* strictly plurisubharmonic.

### Theorem C

Assume that $\Omega$ is a bounded domain in ${\mathbb {C}}^n$, $n\ge 2$. Then the following assertions are equivalent.

1. $\Omega$ is *hyperconvex* in the sense that it admits an exhaustion function that is negative and plurisubharmonic;
2. $\partial \Omega$ has a weak barrier at every point that is plurisubharmonic;
3. $\Omega$ admits an exhaustion function that is negative, smooth and strictly plurisubharmonic;
4. For every $z\in \partial \Omega$, and every Jensen measure $\mu$, which is generated by the cone of functions that are continuous on ${{\bar{\Omega }}}$, and plurisubharmonic on $\Omega$, we have that $\mu$ is carried by $\partial \Omega$.

Historically, the notion of hyperconvexity was introduced by Stehlé in 1974 in connection with the Serre conjecture, and later in 1981 Kerzman and Rosay [39] proved the equivalence of the three first assertions (see also [6]). Kerzman and Rosay also studied which pseudoconvex domains are hyperconvex. We shall not address this question here (see e.g., the introduction of [5] for an up-to-date account of this question). Carlehed et al. [17] showed in 1999 the equivalence between (1) and (4). In connection with Theorems B and C, we would like to mention the inspiring article [8] written by Błocki, the first part of which is a self-contained survey on plurisubharmonic barriers and exhaustion functions in complex domains.

As we mentioned at the beginning of this exposé, the purpose of this paper is to study the geometry of the corresponding notions *B*-regular and hyperconvex domains within the Caffarelli–Nirenberg–Spruck model. More precisely, in Theorem 4.3, we prove what degenerates into Theorem B when $m=n$, and in Theorem 4.1, we prove what is Theorem C in the case $m=n$, except for the corresponding implication $(1)\Rightarrow (3)$. This we prove in Sect. 5 due to the different techniques used, and the length of that proof. In the case when $m=1$, our Theorems 4.3 and 4.1 (together with Theorem 5.4) merge into Theorem A above with $N=2n$.

This article is organized as follows. In Sect. 2, we shall state the necessary definitions and some preliminaries needed for this paper, and then in Sect. 3, we shall prove some basic facts about *m*-hyperconvex domains (Theorem 3.5). From Sect. 3, and Theorem 3.5 we would like to emphasize property (3). Up until now authors have defined *m*-hyperconvex domains to be bounded domains that admit an exhaustion function that is negative, continuous, and *m*-subharmonic. We prove that the assumption of continuity is superfluous. This result is also the starting point of the proof of Theorem 5.4. In Sect. 4, we prove Theorems 4.3 and 4.1, as mentioned above, which correspond to Theorems B and C, respectively. We end this paper by showing that every *m*-hyperconvex domain admits a smooth and strictly *m*-subharmonic exhaustion function (Theorem 5.4; see implication $(1)\Rightarrow (3)$ in Theorem C).

We end this introduction by highlighting an opportunity for future studies related to this paper. As convex analysis and pluripotential theory lives in symbiosis, Trudinger and Wang [60] draw their inspiration from the work of Caffarelli et al., and in 1999 they presented a model that makes it possible to study the transition between convex analysis and potential theory. For further information see e.g., [59–61, 65]. As [63] indicates, further studies of the geometric properties of what could be named *k-convex domains* are of interest. We leave these questions to others.

We want to thank Urban Cegrell, Per-Håkan Lundow, and Håkan Persson for inspiring discussions related to this paper. We are also grateful for the comments and suggestions given by the anonymous referee that helped with the presentation of the final version of this paper.

## Preliminaries

In this section, we shall present the necessary definitions and fundamental facts needed for the rest of this paper. For further information related to potential theory see e.g., [4, 24, 41], and for more information about pluripotential theory see e.g., [22, 40]. We also want to mention the highly acclaimed book written by Hörmander called “*Notions of convexity*” [36]. Abdullaev and Sadullaev [3] have written an article that can be used as an introduction to the Caffarelli–Nirenberg–Spruck model. We recommend also Lu’s doctoral thesis [46]. We would like to point out that *m*-subharmonic functions in the sense of Caffarelli et al. is not equivalent of being subharmonic on *m*-dimensional hyperplanes in ${\mathbb {C}}^n$ studies by others (see e.g., [1, 2]). For other models in connection to plurisubharmonicity see e.g., [31–33].

Let $\Omega \subset {\mathbb {C}}^n$ be a bounded domain, $1\le m\le n$, and define ${\mathbb {C}}_{(1,1)}$ to be the set of (1, 1)-forms with constant coefficients. With this notation we define

$$\begin{aligned} \Gamma _m=\left\{ \alpha \in {\mathbb {C}}_{(1,1)}: \alpha \wedge \beta ^{n-1}\ge 0, \dots , \alpha ^m\wedge \beta ^{n-m}\ge 0 \right\} , \end{aligned}$$

where $\beta =dd^c|z|^2$ is the canonical Kähler form in ${\mathbb {C}}^n$.

### Definition 2.1

Assume that $\Omega \subset {\mathbb {C}}^n$ is a bounded domain, and let *u* be a subharmonic function defined in $\Omega$. Then we say that *u* is *m-subharmonic*, $1\le m\le n$, if the following inequality holds

$$\begin{aligned} dd^cu\wedge \alpha _1\wedge \dots \wedge \alpha _{m-1}\wedge \beta ^{n-m}\ge 0, \end{aligned}$$

in the sense of currents for all $\alpha _1,\ldots ,\alpha _{m-1}\in \Gamma _m$. With ${\mathcal {SH}}_m(\Omega )$ we denote the set of all *m*-subharmonic functions defined on $\Omega$. We say that a function *u* is *strictly m-subharmonic* if it is *m*-subharmonic on $\Omega$, and for every $p \in \Omega$ there exists a constant $c_p >0$ such that $u(z)-c_p |z| ^2$ is *m*-subharmonic in a neighborhood of *p*.

### Remark

From Definition 2.1 it follows that

$$\begin{aligned} \mathcal {PSH}={\mathcal {SH}}_n \subset \cdots \subset {\mathcal {SH}}_1 ={\mathcal {SH}}. \end{aligned}$$

In Theorem 2.2, we give a list of well-known properties that *m*-subharmonic functions enjoy.

### Theorem 2.2

Assume that $\Omega \subset {\mathbb {C}}^n$ is a bounded domain, and $1\le m\le n$. Then we have that

1. If $u,v\in {\mathcal {SH}}_m(\Omega )$, then $su+tv\in {\mathcal {SH}}_m(\Omega )$, for constants $s,t\ge 0$;
2. If $$u,v\in {\mathcal {SH}}_m(\Omega )$$, then $$\max \{u,v\}\in {\mathcal {SH}}_m(\Omega )$$;
3. If $\{u_{\alpha }\}$ is a locally uniformly bounded family of functions from ${\mathcal {SH}}_m(\Omega )$, then the upper semicontinuous regularization
  $$\begin{aligned} \left( \sup _{\alpha }u_{\alpha }\right) ^* \end{aligned}$$
  defines a *m*-subharmonic function;
4. If $\{u_j\}$ is a sequence of functions in ${\mathcal {SH}}_m(\Omega )$ such that $u_j \searrow u$ and there is a point $z \in \Omega$ such that $u(z) > - \infty$, then $u \in {\mathcal {SH}}_m(\Omega )$;
5. If $u\in {\mathcal {SH}}_m(\Omega )$ and $\gamma :{\mathbb {R}}\rightarrow {\mathbb {R}}$ is a convex and nondecreasing function, then $\gamma \circ u\in {\mathcal {SH}}_m(\Omega )$;
6. If $u\in {\mathcal {SH}}_m(\Omega )$, then the standard regularization given by the convolution $u\star \rho _{\varepsilon }$ is *m*-subharmonic in $\{z\in \Omega :{\text {dist}}(z,\partial \Omega )>\varepsilon \}$. Here we have that
  $$\begin{aligned} \rho _{\varepsilon }=\varepsilon ^{-2n}\rho \left( \frac{z}{\varepsilon }\right) , \end{aligned}$$
  $\rho :{\mathbb {R}}_+\rightarrow {\mathbb {R}}_+$ is a smooth function such that $\rho (z)=\rho (|z|)$ and
  $$\begin{aligned} \rho (t)= {\left\{ \begin{array}{ll} \frac{C}{(1-t)^2}\exp \left( {\frac{1}{t-1}}\right) &{} \text { when } t\in [0,1]\\ 0 &{} \text { when } t\in (1,\infty ), \end{array}\right. } \end{aligned}$$
  where *C* is a constant such that $\int _{{\mathbb {C}}^n}\rho (|z|^2)\beta ^n=1$;
7. If $\omega \Subset \Omega$, $u\in {\mathcal {SH}}_m(\Omega )$, $v\in {\mathcal {SH}}_m(\omega )$, and $\varlimsup _{z\rightarrow w}v(z)\le u(w)$ for all $w\in \partial \omega$, then the function defined by
  $$\begin{aligned} \varphi = {\left\{ \begin{array}{ll} u, \, \text{ on } \, \Omega \setminus \omega ,\\ \max \{u,v\}, \; \text{ on } \, \omega , \end{array}\right. } \end{aligned}$$
  is *m*-subharmonic on $\Omega$;

We shall need several different envelope constructions. We have gathered their definitions and notations in Definition 2.3.

### Definition 2.3

Assume that $\Omega \subset {\mathbb {C}}^n$ is a bounded domain, and $1\le m\le n$.

- (a) For $f\in {\mathcal {C}}({{\bar{\Omega }}})$ we define
  $$\begin{aligned} \mathbf{S }_f(z)=\sup \left\{ v(z): v\in \mathcal {SH}_m(\Omega ), v\le f\right\} , \end{aligned}$$
  and similarly
  $$\begin{aligned} \mathbf{S }^c_f(z)=\sup \left\{ v(z): v\in \mathcal {SH}_m(\Omega )\cap {\mathcal {C}}({{\bar{\Omega }}}), v\le f\right\} . \end{aligned}$$
- (b) If instead $f\in {\mathcal {C}}(\partial \Omega )$, then we let
  $$\begin{aligned} \mathbf{S }_f(z)=\sup \left\{ v(z): v\in \mathcal {SH}_m(\Omega ), v^*\le f\, \text{ on } \, \partial \Omega \right\} , \end{aligned}$$
  and
  $$\begin{aligned} \mathbf{S }^c_f(z)=\sup \left\{ v(z): v\in \mathcal {SH}_m(\Omega )\cap {\mathcal {C}}({{\bar{\Omega }}}), v\le f\, \text{ on } \, \partial \Omega \right\} . \end{aligned}$$

### Remark

Assume that $$m=1$$. If $$\Omega \subset {\mathbb {C}}^n$$$$(\cong {\mathbb {R}}^{2n})$$ is a regular domain in the sense of Theorem A, and if $$f\in {\mathcal {C}}(\partial \Omega )$$, then $${\text {PWB}}_f$$ (defined also in Theorem A) is the unique harmonic function on $$\Omega $$, continuous on $${{\bar{\Omega }}}$$, such that $${\text {PWB}}_f=f$$ on $$\partial \Omega $$. Therefore, we have that $$\mathbf{S }_f(z)=\mathbf{S }_{{\text {PWB}}_f}(z)$$, and $$\mathbf{S }^c_f(z)=\mathbf{S }^c_{{\text {PWB}}_f}(z)$$.

In Definition 2.4, we state the definition of relative extremal functions in our setting.

### Definition 2.4

Assume that $E\Subset \Omega$ is an open subset such that $\Omega \setminus {{\bar{E}}}$ is a regular domain in the sense of Theorem A. Then we make the following definitions

$$\begin{aligned} \mathbf{S }_E(z)=\sup \left\{ v(z): v\in \mathcal {SH}_m(\Omega ), v\le -1\, \text{ on } \, E, v\le 0 \right\} , \end{aligned}$$

and

$$\begin{aligned} \mathbf{S }^c_E(z)=\sup \left\{ v(z): v\in \mathcal {SH}_m(\Omega )\cap {\mathcal {C}}({{\bar{\Omega }}}), v\le -1\, \text{ on } \, E, v\le 0 \right\} . \end{aligned}$$

### Remark

From well-known potential theory, we have that if $h_E$ is the unique harmonic function defined on $\Omega \setminus {{\bar{E}}}$, continuous on ${{\bar{\Omega }}}\setminus E$, $h_E=0$ on $\partial \Omega$, $h_E=-1$ on $\partial E$, and if we set

$$\begin{aligned} \mathbf{H }_E(z)= {\left\{ \begin{array}{ll} h_E(z) &{} \text { if } z\in {{\bar{\Omega }}} \setminus E \\ -1&{} \text { if } z\in E, \end{array}\right. } \end{aligned}$$

then we have that $\mathbf{S }_E(z)=\mathbf{S }_{\mathbf{H }_E}(z)$. To see it assume that $v\in \mathcal {SH}_m(\Omega )$, $v\le -1$ on *E*, $v\le 0$, then $v\le h_E$ on ${\bar{\Omega }}\setminus E$ and therefore $v\le \mathbf{H }_E$, which means that $\mathbf{S }_E\le \mathbf{S }_{\mathbf{H }_E}$. On the other hand since $\mathbf{S }_{\mathbf{H }_E}^*\in \mathcal {SH}_m(\Omega )$ and $\mathbf{S }_{\mathbf{H }_E}^*\le -1$ on *E* which implies that

$$\begin{aligned} \mathbf{S }_{\mathbf{H }_E}^*\le \mathbf{S }_E\le \mathbf{S }_{\mathbf{H }_E}\le \mathbf{S }_{\mathbf{H }_E}^*. \end{aligned}$$

Błocki’s generalization of Walsh’s celebrated Theorem [62], and an immediate consequence will be needed as well.

### Theorem 2.5

Let $\Omega$ be a bounded domain in ${\mathbb {C}}^n$, and let $f\in {\mathcal {C}}({{\bar{\Omega }}})$. If for all $w\in \partial \Omega$ we have that $\lim _{z\rightarrow w}\mathbf{S }_f(z)=f(w)$, then $\mathbf{S }_f\in {\mathcal {SH}}_m(\Omega )\cap {\mathcal {C}}({{\bar{\Omega }}})$.

### Proof

See Proposition 3.2 in [9]. $\square$

A direct consequence of Theorem 2.5 is the following.

### Corollary 2.6

Let $\Omega$ be a bounded domain in ${\mathbb {C}}^n$, and let $f\in {\mathcal {C}}({\bar{\Omega }})$. If for all $w\in \partial \Omega$ we have that $\lim _{z\rightarrow w}\mathbf{S }^c_f(z)=f(w)$, then $\mathbf{S }^c_f=\mathbf{S }_f\in {\mathcal {SH}}_m(\Omega )\cap {\mathcal {C}}({\bar{\Omega }})$.

### Proof

First note that

$$\begin{aligned} \mathbf{S }^c_f\le \mathbf{S }_f\le f. \end{aligned}$$

Therefore, if

$$\begin{aligned} \lim _{z\rightarrow w}\mathbf{S }^c_f(z)=f(w), \end{aligned}$$

holds for all $w\in \partial \Omega$, then

$$\begin{aligned} \lim _{z\rightarrow w}\mathbf{S }_f(z)=f(w). \end{aligned}$$

Hence, by Theorem 2.5, we get that $\mathbf{S }_f\in {\mathcal {SH}}_m(\Omega )\cap {\mathcal {C}}({\bar{\Omega }})$, which gives us that $\mathbf{S }_f\le \mathbf{S }^c_f$. Thus, $\mathbf{S }_f=\mathbf{S }^c_f$. $\square$

In Sect. 4, we shall make use of techniques from Choquet theory, in particular Jensen measures w.r.t. the cone ${\mathcal {SH}}_m(\Omega ) \cap {\mathcal {C}}({\bar{\Omega }})$ of continuous functions. This is possible since ${\mathcal {SH}}_m(\Omega ) \cap {\mathcal {C}}({\bar{\Omega }})$ contains the constant functions and separates points in ${\mathcal {C}}({\bar{\Omega }})$. Our inspiration can be traced back to the works mentioned in the introduction, but maybe more to [17] and [35].

### Definition 2.7

Let $\Omega$ be a bounded domain in ${\mathbb {C}}^n$, and let $\mu$ be a non-negative regular Borel measure defined on $\bar{\Omega }$. We say that $\mu$ is a *Jensen measure with barycenter*$z_0\in \bar{\Omega }$*w.r.t.*${\mathcal {SH}}_m(\Omega ) \cap {\mathcal {C}}({\bar{\Omega }})$ if

$$\begin{aligned} u(z_0)\le \int _{\bar{\Omega }} u \, d\mu \qquad \qquad \text {for all } u \in {\mathcal {SH}}_m(\Omega ) \cap {\mathcal {C}}({\bar{\Omega }})\, . \end{aligned}$$

The set of such measures will be denoted by ${\mathcal {J}}_{z_0}^m$. Furthermore, the *Jensen boundary* w.r.t. ${\mathcal {J}}_{z_0}^m$ is defined as

$$\begin{aligned} \partial _{{\mathcal {J}}^m}=\left\{ z\in \bar{\Omega }: {\mathcal {J}}_{z}^m=\{\delta _z\} \right\} . \end{aligned}$$

### Remark

The Jensen boundary is another name for the Choquet boundary w.r.t. a given class of Jensen measures. For further information see e.g., [12, 48].

### Remark

There are many different spaces of Jensen measures introduced throughout the literature. Caution is advised.

The most important tool in working with Jensen measures is the Edwards’ duality theorem that origins from [25]. We only need a special case formulated in Theorem 2.8. For a proof, and a discussion, of Edwards’ theorem see [69] (see also [20, 21, 55]).

### Theorem 2.8

(Edwards’ Theorem). Let $\Omega$ be a bounded domain in ${\mathbb {C}}^n$, and let *g* be a real-valued lower semicontinuous function defined on ${\bar{\Omega }}$. Then for every $z\in \bar{\Omega }$ we have that

$$\begin{aligned} \mathbf{S }_g^c(z)=\sup \{v(z):v \in {\mathcal {SH}}_m(\Omega ) \cap {\mathcal {C}}({\bar{\Omega }}), v \le g \}=\inf \left\{ \int g \, d\mu : \mu \in {\mathcal {J}}_z^m\right\} . \end{aligned}$$

We end this section with a convergence result.

### Theorem 2.9

Assume that $\Omega$ is a domain in ${\mathbb {C}}^n$, and let $\{z_n\} \subset {\bar{\Omega }}$ be a sequence of points converging to $z\in {\bar{\Omega }}$. Furthermore, for each *n*, let $\mu _n \in {\mathcal {J}}_{z_n}^m$. Then there exists a subsequence $\{\mu _{n_j}\}$, and a measure $\mu \in {\mathcal {J}}_z^m$ such that $\{\mu _{n_j}\}$ converges in the weak-$^*$ topology to $\mu$.

### Proof

The Banach–Alaoglu theorem says that the space of probability measures defined on ${\bar{\Omega }}$ is compact when equipped with the weak-$^*$ topology. This means that there is a subsequence $\{\mu _{n_j}\}$ that converges to a probability measure $\mu$. It remains to show that $\mu \in {\mathcal {J}}_z^m$. Take $u \in {\mathcal {SH}}_m(\Omega )\cap {\mathcal {C}}({\bar{\Omega }})$ then

$$\begin{aligned} \int u \, d\mu = \lim _j \int u \, d\mu _{n_j} \ge \lim _ju(z_j)=u(z), \end{aligned}$$

hence $$\mu \in {\mathcal {J}}_z^m$$. $$\square $$

## Basic Properties of *m*-Hyperconvex Domains

The aim of this section is to introduce *m*-hyperconvex domains (Definition 3.1) within the Caffarelli–Nirenberg–Spruck model, and prove Theorem 3.5 . If $m=1$, then the notion will be the same as regular domains (see assertion (4) in Theorem A in the introduction), and if $m=n$ then it is the same as hyperconvex domains (see (1) in Theorem C).

### Definition 3.1

Let $\Omega$ be a bounded domain in ${\mathbb {C}}^n$. We say that $\Omega$ is *m-hyperconvex* if it admits an exhaustion function that is negative and *m*-subharmonic.

Traditionally, in pluripotential theory, the exhaustion functions are assumed to be bounded. That assumption is obviously superfluous in Definition 3.1. Even though it should be mentioned once again that up until now authors have defined *m*-hyperconvex domains to be bounded domains that admit an exhaustion function that is negative, *continuous*, and *m*-subharmonic. We prove below in Theorem 3.5 that the assumption of continuity is not necessary. Before continuing with Theorem 3.5 let us demonstrate the concept of *m*-hyperconvexity in the following two examples. Example 3.2 demonstrates that Hartogs’ triangle is 1-hyperconvex, but not 2-hyperconvex.

### Example 3.2

Set

$$\begin{aligned} \Omega =\{(z,w) \in {\mathbb {C}}^2: |z |< | w | <1\}. \end{aligned}$$

This is Hartogs’ triangle, and it is not hyperconvex (Proposition 1 in [26]), but it is a regular domain. In other words, it is not 2-hyperconvex, but it is 1-hyperconvex. It is easy to see that

$$\begin{aligned} \varphi (z,w)=\max \big \{\log |w|,|z|^2-|w|^2\big \} \end{aligned}$$

is a negative, subharmonic (1-subharmonic) exhaustion function for $\Omega$. $\square$

In Example 3.3, we construct a domain in ${\mathbb {C}}^3$ that is 2-hyperconvex, but not 3-hyperconvex.

### Example 3.3

For a given integer $1\le k\le n$, let $\varphi _k$ be the function defined on ${\mathbb {C}}^n$ by

$$\begin{aligned} \varphi _k(z_1,\ldots ,z_n)=|z_1|^2+\ldots +|z_{n-1}|^2+\left( 1-\frac{n}{k}\right) |z_n|^2. \end{aligned}$$

Then we have that $\varphi _k$ is *m*-subharmonic function if, and only if, $m\le k$. Let us now consider the following domain:

$$\begin{aligned} \Omega _k=\left\{ (z_1,\dots ,z_n)\in {\mathbb {C}}^n: |z_1|<1, \dots , |z_n|<1, \varphi _k(z)<1 \right\} . \end{aligned}$$

This construction yields that $\Omega _k$ is a balanced Reinhardt domain that is not pseudoconvex (see e.g., Theorem 1.11.13 in [38]). Furthermore, we have that $\Omega _k$ is *k*-hyperconvex, since

$$\begin{aligned} u(z_1,\ldots ,z_n)=\max \{|z_1|,\dots ,|z_n|, \varphi _k(z)\}-1\, \end{aligned}$$

is a *k*-subharmonic exhaustion function. In particular, we get that for $n=3$, and $k=2$, the domain $\Omega _2\subset {\mathbb {C}}^3$ is 2-hyperconvex but not 3-hyperconvex. $\square$

We shall need the following elementary lemma. For completeness we include a proof.

### Lemma 3.4

Let $\chi :(-\infty ,0)\rightarrow (0,\infty )$ be an increasing and convex function. Then for any $a<b<0$ and $x<0$ we have that

$$\begin{aligned} \chi (b+x)-\chi (a+x)\le \chi (b)-\chi (a). \end{aligned}$$

### Proof

For $x<0$, let us define

$$\begin{aligned} \theta (x)=\int _{a+x}^{b+x}\chi '_r(t)\,dt, \end{aligned}$$

where $\chi '_r$ denotes the right derivative of $\chi$ (which exists since $\chi$ is convex). By our assumptions, we know that $\chi '_r$ is a non-negative and nondecreasing function. Therefore, $\theta$ is also nondecreasing, and then for any $x<0$ we have that

$$\begin{aligned} \chi (b+x)-\chi (a+x)=\theta (x)\le \theta (0)=\chi (b)-\chi (a) . \end{aligned}$$

$$\square $$

The aim of this section is to prove the following theorem, especially property (3).

### Theorem 3.5

Assume that $\Omega$, $\Omega _1$, and $\Omega _2$ are bounded *m*-hyperconvex domains in ${\mathbb {C}}^n$, $n\ge 2$, $1\le m\le n$. Then we have the following.

1. If $\Omega _1 \cap \Omega _2$ is connected, then the domain $\Omega _1 \cap \Omega _2$ is *m*-hyperconvex in ${\mathbb {C}}^n$.
2. The domain $\Omega _1 \times \Omega _2$ is *m*-hyperconvex in ${\mathbb {C}}^{2n}$.
3. The domain $\Omega$ admits a negative exhaustion function that is strictly *m*-subharmonic on $\Omega$, and continuous on ${\bar{\Omega }}$.
4. If $\Omega$ is a priori only a bounded domain in ${\mathbb {C}}^n$ such that for every $z\in \partial \Omega$ there exists a neighborhood $U_z$ such that $\Omega \cap U_z$ is *m*-hyperconvex, then $\Omega$ is *m*-hyperconvex.

### Proof

*Part (1)* For each $j=1,2$, assume that $\psi _j \in {\mathcal {SH}}_m(\Omega _j)$ is a negative exhaustion function for the *m*-hyperconvex domain $\Omega _j$, $j=1,2$. Then $\max \{\psi _1,\psi _2\} \in {\mathcal {SH}}_m(\Omega _1\cap \Omega _2)$ is a negative exhaustion function for $\Omega _1 \cap \Omega _2$. Thus, $\Omega _1 \cap \Omega _2$ is *m*-hyperconvex in ${\mathbb {C}}^n$.

*Part (2)* This part is concluded by defining a negative exhaustion function by

$$\begin{aligned} \psi (z_1,z_2)=\max \{\psi _1(z_1),\psi _2(z_2)\} \in {\mathcal {SH}}_m(\Omega _1\times \Omega _2). \end{aligned}$$

*Part (3)* The proof of this part is inspired by [19]. First, we shall prove that there exists a negative and continuous exhaustion function. We know that $\Omega$ always admits a bounded, negative, exhaustion function $\varphi \in {\mathcal {SH}}_m(\Omega )$. Fix $w\in \Omega$ and $r>0$ such that $B(w,r)\Subset \Omega$, and note that there exists a constant $M>0$ such that

$$\begin{aligned} M\varphi \le \mathbf{H }_{B(w,r)} \end{aligned}$$

(the definition of $\mathbf{H }_{B(w,r)}$ is in the remark after Definition 2.4). This construction implies that

$$\begin{aligned} 0=\lim _{z\rightarrow \partial \Omega }M\varphi (z)\le \lim _{z\rightarrow \partial \Omega }\mathbf{S }_{\mathbf{H }_{B(w,r)}}(z)\le \lim _{z\rightarrow \partial \Omega }\mathbf{H }_{B(w,r)}(z)=0. \end{aligned}$$

Thanks to the generalized Walsh theorem (Theorem 2.5) we have that

$$\begin{aligned} \mathbf{S }_{\mathbf{H }_{B(w,r)}}=\mathbf{S }_{B(w,r)}\in {\mathcal {SH}}_m(\Omega )\cap {\mathcal {C}}({\bar{\Omega }}), \end{aligned}$$

and that $\mathbf{S }_{B(w,r)}$ is a continuous exhaustion function.

Next, we shall construct a continuous *strictlym*-subharmonic exhaustion function for $\Omega$. From the first part of this theorem, we know that there is a negative and continuous exhaustion function $u \in {\mathcal {SH}}_m(\Omega )\cap {\mathcal {C}}({\bar{\Omega }})$ for $\Omega$. Choose $M>0$ such that $|z|^2-M \le -1$ on $\Omega$, and define

$$\begin{aligned} \psi _j(z)=\max \left\{ u(z),\frac{|z|^2-M}{j}\right\} . \end{aligned}$$

Then $\psi _j \in {\mathcal {SH}}_m(\Omega )\cap {\mathcal {C}}({\bar{\Omega }})$, $\psi _j|_{\partial \Omega }=0$, and $\psi _j <0$ on $\Omega$. If we now let

$$\begin{aligned} a_j=\frac{1}{2^j}\frac{1}{\max \{\sup (-\psi _j),1\}}, \qquad \text {and} \qquad \psi =\sum _{j=1}^{\infty }a_j\psi _j, \end{aligned}$$

then $\psi ^k=\sum _{j=1}^{k}a_j\psi _j$ defines a decreasing sequence of continuous *m*-subharmonic functions on defined $\Omega$. We can conclude that $\psi \in {\mathcal {SH}}_m(\Omega )$, since $\psi (z)> -\infty$ for $z \in \Omega$. The continuity of $\psi$ is obtained by the Weierstrass *M*-test. To see that $\psi$ is strictly *m*-subharmonic, note that if $\omega \Subset \Omega$, then there exists an index $j_\omega$ such that on $\omega$ we have that

$$\begin{aligned} \psi _j=\frac{|z|^2-M}{j} \qquad \text { for all } j>j_\omega . \end{aligned}$$

This gives us that

$$\begin{aligned} \psi =\sum _{j=1}^{j_\omega }a_j \psi _j+\sum _{j=j_\omega +1}^{\infty }a_j \frac{|z|^2-M}{j}. \end{aligned}$$

Since $\frac{|z|^2-M}{j}$ is strictly plurisubharmonic, and therefore strictly *m*-subharmonic, we have that $\psi$ is strictly *m*-subharmonic on $\Omega$. Finally, $\psi$ is an exhaustion function for $\Omega$, since $\psi _j|_{\partial \Omega }=0$ for all *j*.

*Part (4)* The idea of the proof of this part is from [7]. By the assumption there are neighborhoods $U_{z_1},\dots ,U_{z_N}$ such that $\partial \Omega \subset \bigcup _{j=1}^NU_{z_j}$, and each $U_{z_j}\cap \Omega$ is *m*-hyperconvex. Let $u_j:\Omega \rightarrow [-1,0]$ be a negative and continuous *m*-subharmonic exhaustion function for $U_{z_j}\cap \Omega$. Let $V_j\Subset U_{z_j}$ be such that $\partial \Omega \subset \bigcup _{j=1}^NV_{j}$. For $x<0$, we then define the following continuous functions

$$\begin{aligned} \begin{aligned} \beta (x)&=\max \left\{ u_j(z): z\in {{\bar{V}}}_j\cap \Omega , j=1,\dots , N, {\text {dist}}(z,\partial \Omega )\le -x \right\} , \\ \alpha (x)&=\min \left\{ u_j(z): z\in {{\bar{V}}}_j\cap \Omega , j=1,\dots , N, {\text {dist}}(z,\partial \Omega )\le -x \right\} . \end{aligned} \end{aligned}$$

From these definitions, it follows that $\alpha \le \beta$, and $\lim _{x\rightarrow 0^-}\alpha (x)=0$. Therefore, there exists a convex, increasing function $\chi :(-\infty ,0)\rightarrow (0,\infty )$ such that $\lim _{x\rightarrow 0^-}\chi (x)=\infty$, and $\chi \circ \beta \le \chi \circ \alpha +1$ (see e.g., Lemma A2.4. in [7]). Hence,

$$\begin{aligned} |\chi \circ u_j-\chi \circ u_k|\le 1 \, \text{ on } \, V_j\cap V_k\cap \Omega . \end{aligned}$$

For any $\varepsilon >0$ we have that

3.1 $$\begin{aligned} |\chi (u_j(z)-\varepsilon )-\chi (u_k(z)-\varepsilon )|\le 1 \, \text{ for } \, z\in V_j\cap V_k\cap \Omega , \end{aligned}$$

since $\chi$ is an increasing and convex function (see Lemma 3.4). Next, let $V'_j\Subset V_j$, $j=1,\dots ,N$, be such that ${\bar{\Omega }}\setminus V\subset \bigcup _{j=1}^NV_j'$, for some open set $V\Subset \Omega$. For each *j*, take a smooth function $\varphi _j$ such that ${\text {supp}}(\varphi _j)\subset V_j$, $0\le \varphi _j\le 1$, and $\varphi _j=1$ on a neighborhood of ${{\bar{V}}}_j'$. Furthermore, there are constants $M_1,M_2>0$ such that $|z|^2-M_1\le 0$ on $\Omega$, and such that the functions $\varphi _j+M_2(|z|^2-M_1)$ are *m*-subharmonic for $j=1,\dots ,N$. Let us define

$$\begin{aligned} v_{j,\varepsilon }(z)=\chi (u_j(z)-\varepsilon )+\varphi _j(z)-1+M_2(|z|^2-M_1)\, . \end{aligned}$$

From (3.1) it then follows that

3.2 $$\begin{aligned} v_{j,\varepsilon }\le v_{k,\varepsilon } \, \text{ on } \text{ a } \text{ neighborhood } \text{ of } \, \partial V_j\cap {{\bar{V}}}_k'\cap \Omega . \end{aligned}$$

Take yet another constant *c* such that

3.3 $$\begin{aligned} \sup \left\{ u_j(z): z\in V\cap V_j, j=1,\dots , N\right\} \,< c<0\, , \end{aligned}$$

and define

$$\begin{aligned} v_{\varepsilon }(z)=\max \left\{ v_{j,\varepsilon }(z),\chi (c)-1+M_2(|z|^2-M_1)\right\} . \end{aligned}$$

By (3.2), and (3.3), it follows that $v_{\varepsilon }$ is a well-defined *m*-subharmonic function defined on $\Omega$. Finally note that, for $c<-\varepsilon$, the following function

$$\begin{aligned} \psi _{\varepsilon }(z)=\frac{v_{\varepsilon }(z)}{\chi (-\varepsilon )}-1 \end{aligned}$$

is *m*-subharmonic, and $\psi _{\varepsilon }\le 0$ on $\Omega$. For $z\in \partial \Omega \cap V_j'$, we have that

$$\begin{aligned} u_j(z)=0\qquad \text { and }\qquad \varphi _j(z)=1, \end{aligned}$$

hence

3.4 $$\begin{aligned} \psi _{\varepsilon }(z)\ge \frac{v_{j,\varepsilon }(z)}{\chi (-\varepsilon )}-1=\frac{\chi (-\varepsilon )+M_2(|z|^2-M_1)}{\chi (-\varepsilon )}-1\ge -\frac{M_1M_2}{\chi (-\varepsilon )}. \end{aligned}$$

In addition, it holds that

3.5 $$\begin{aligned} \psi _{\varepsilon }(z)\le \frac{\chi (c)-1}{\chi (-\varepsilon )}-1, \, z\in V\setminus \bigcup _{j=1}^NV_j. \end{aligned}$$

Now fix a ball $B(z,r)\Subset V\setminus \bigcup _{j=1}^NV_j$. From (3.4), (3.5), and the fact that

$$\begin{aligned} \lim _{x\rightarrow 0^-}\chi (x)=\infty \end{aligned}$$

we have that

$$\begin{aligned} (\sup _{\varepsilon }\psi _{\varepsilon })^*\le \mathbf{S }_{B(z,r)} \end{aligned}$$

(see Definition 2.4). Thus,

$$\begin{aligned} \lim _{\xi \rightarrow \partial \Omega }\mathbf{S }_{B(z,r)}(\xi )=0. \end{aligned}$$

Theorem 2.5 (generalized Walsh’s theorem) gives us that

$$\begin{aligned} \mathbf{S }_{B(z,r)}\in \mathcal {SH}_m(\Omega )\cap {\mathcal {C}}({\bar{\Omega }}), \end{aligned}$$

and that $\mathbf{S }_{B(z,r)}$ is the desired exhaustion function for $\Omega$. This ends the proof of Part (4), and this theorem. $\square$

### Remark

Assume that $\Omega$ is bounded *m*-hyperconvex domain, and $E\Subset \Omega$ is an open subset such that $\Omega \setminus {{\bar{E}}}$ is a regular domain in the sense of Theorem A. Then we have that

1. $$\displaystyle {\mathbf{S }_E^c(z)=\mathbf{S }_{\mathbf{H }_E}^c(z)}$$, and
2. $$\displaystyle {\mathbf{S }_E=\mathbf{S }_E^c=\mathbf{S }_{\mathbf{H }_E}^c=\mathbf{S }_{\mathbf{H }_E}}$$.

To see that (1) holds, first note that it is clear that $\mathbf{S }_E^c(z)\le \mathbf{S }_{\mathbf{H }_E}^c(z)$. On the other hand, since $\Omega$ is a *m*-hyperconvex domain it can be proved as in part (3) of Theorem 3.5 that $\mathbf{S }_{\mathbf{H }_E}^c\in \mathcal {SH}_m(\Omega )\cap {\mathcal {C}}({\bar{\Omega }})$. Therefore, $\mathbf{S }_E^c(z)\ge \mathbf{S }_{\mathbf{H }_E}^c(z)$. Property (2) follows from Theorem 2.5 together with the remark after Definition 2.4.

## The Geometry of *m*-Regular Domains

In this section, we shall investigate the geometry of the corresponding notions of *B*-regular and hyperconvex domains within the Caffarelli–Nirenberg–Spruck model. More precisely, in Theorem 4.3, we prove what degenerates into Theorem B when $m=n$, and in Theorem 4.1, we prove what is Theorem C in the case $m=n$.

### Theorem 4.1

Assume that $\Omega$ is a bounded domain in ${\mathbb {C}}^n$, $n\ge 2$, $1\le m\le n$. Then the following assertions are equivalent.

1. $\Omega$ is *m*-hyperconvex in the sense of Definition 3.1;
2. $\partial \Omega$ has a weak barrier at every point that is *m*-subharmonic;
3. $\Omega$ admits an exhaustion function that is negative, smooth and strictly *m*-subharmonic; and
4. For every $z\in \partial \Omega$, and every $\mu \in {\mathcal {J}}_z^m$, we have that ${\text {supp}} (\mu ) \subseteq \partial \Omega$.

### Proof

The implications $(1)\Rightarrow (2)$, and $(3)\Rightarrow (1)$ are trivial. The implication $(1)\Rightarrow (3)$ is postponed to Theorem 5.4 in Sect. 5.

$(2)\Rightarrow (1):$ Let $w\in \Omega$ and $r>0$ be such that the ball $B(w,r)\Subset \Omega$. Then by assumption, we have that for every $z\in \partial \Omega$ there exists a weak barrier $u_z$ at *z* that is *m*-subharmonic. Since there exists a constant $M_z>0$ such that

$$\begin{aligned} M_zu_z\le \mathbf{S }_{B(w,r)} \end{aligned}$$

it follows that

$$\begin{aligned} \lim _{\xi \rightarrow \partial \Omega }\mathbf{S }_{B(w,r)}(\xi )=0. \end{aligned}$$

Thanks to the generalized Walsh theorem (Theorem 2.5), we know that $\mathbf{S }_{B(w,r)}\in \mathcal {SH}_m(\Omega )\cap {\mathcal {C}}({\bar{\Omega }})$. Hence, $\mathbf{S }_{B(w,r)}$ is an exhaustion function for $\Omega$.

$(1)\Rightarrow (4):$ Assume that $\Omega$ is *m*-hyperconvex, and that $u \in {\mathcal {SH}}_m(\Omega )\cap {\mathcal {C}}({\bar{\Omega }})$ is an exhaustion function for $\Omega$. If $z \in \partial \Omega$, and $\mu \in {\mathcal {J}}_z^m$, then

$$\begin{aligned} 0=u(z)\le \int u \, d\mu \le 0. \end{aligned}$$

This implies that ${\text {supp}} (\mu ) \subseteq \partial \Omega$, since $u <0$ on $\Omega$.

$(4)\Rightarrow (1):$ Suppose that ${\text {supp}}(\mu ) \subset \partial \Omega$ for all $\mu \in {\mathcal {J}}_z^m$, $z \in \partial \Omega$. Let $w\in \Omega$, $r>0$, be such that the ball $B(w,r)\Subset \Omega$, and let

$$\begin{aligned} \mathbf{S }^c_{\overline{B(w,r)}}(z)= \sup \{\varphi (z): \varphi \in {\mathcal {SH}}_m(\Omega )\cap {\mathcal {C}}({\bar{\Omega }}), \varphi \le 0, \varphi \le -1 \ \text {on} \ \overline{B(w,r)}\}\,. \end{aligned}$$

From Edwards’ theorem (Theorem 2.8), it follows that

$$\begin{aligned} \mathbf{S }^c_{\overline{B(w,r)}}(z)=\inf \left\{ \int -\chi _{\overline{B(w,r)}} \, d\mu : \mu \in {\mathcal {J}}_z^m\right\} =-\sup \left\{ \mu (\overline{B(w,r)}): \mu \in {\mathcal {J}}_z^m\right\} . \end{aligned}$$

We shall now prove that

$$\begin{aligned} \lim _{\xi \rightarrow \partial \Omega }\mathbf{S }^c_{\overline{B(w,r)}}(\xi )=0, \end{aligned}$$

and this shall be done with a proof by contradiction. Assume the contrary, i.e., that there is a point $z \in \partial \Omega$ such that

$$\begin{aligned} \varliminf _{\xi \rightarrow z}\mathbf{S }^c_{\overline{B(w,r)}}(\xi )<0. \end{aligned}$$

Then we can find a sequence $\{z_n\}$, that converges to *z*, and

$$\begin{aligned} \mathbf{S }^c_{\overline{B(w,r)}}(z_n)<-\varepsilon \qquad \text { for every } n. \end{aligned}$$

We can find corresponding measures $\mu _n \in {\mathcal {J}}_{z_n}^m$ such that $\mu _n(\overline{B(w,r)})>\varepsilon$. By passing to a subsequence, Theorem 2.9 gives us that we can assume that $\mu _n$ converges in the weak-$^*$ topology to a measure $\mu \in {\mathcal {J}}_z^m$. Lemma 2.3 in [17], implies then that

$$\begin{aligned} \mu (\overline{B(w,r)})=\int \chi _{\overline{B(w,r)}} \, d\mu \ge \varlimsup _{n \rightarrow \infty } \int \chi _{\overline{B(w,r)}} \, d\mu _n =\varlimsup _{n \rightarrow \infty } \mu _n(\overline{B(w,r)}) >\varepsilon \ge 0. \end{aligned}$$

This contradicts the assumption that $\mu \in {\mathcal {J}}_z^m$ only has support on the boundary. Hence, Corollary 2.6 gives us that

$$\begin{aligned} \mathbf{S }^c_{B(w,r)} \in \mathcal {SH}_m(\Omega )\cap {\mathcal {C}}({\bar{\Omega }}), \end{aligned}$$

and that $\mathbf{S }^c_{B(w,r)}$ is an exhaustion function for $\Omega$. Thus, $\Omega$ is *m*-hyperconvex. $\square$

Before we can start with the proof of Theorem 4.3, we need the following corollary.

### Corollary 4.2

Let $\Omega$ be a bounded *m*-hyperconvex domain in ${\mathbb {C}}^n$, and let $f \in {\mathcal {C}}(\partial \Omega )$. Then there exists a function $u \in {\mathcal {SH}}_m(\Omega ) \cap {\mathcal {C}}({\bar{\Omega }})$ such that $u=f$ on $\partial \Omega$ if, and only if,

$$\begin{aligned} f(z)=\inf \left\{ \int f \, d\mu : \mu \in {\mathcal {J}}_z^m\right\} \qquad \text { for all } z \in \partial \Omega . \end{aligned}$$

### Proof

Assume that $f \in {\mathcal {C}}(\partial \Omega )$, and that $u \in {\mathcal {SH}}_m(\Omega )\cap {\mathcal {C}}({\bar{\Omega }})$ is such that $u=f$ on $\partial \Omega$. Let $z \in \partial \Omega$, and $\mu \in {\mathcal {J}}_z^m$, then we have that

$$\begin{aligned} f(z)=u(z) \le \int u \, d\mu , \end{aligned}$$

which, together with Theorem 4.1, imply that

$$\begin{aligned} f(z) \le \inf \left\{ \int u \, d\mu : \mu \in {\mathcal {J}}_z^m\right\} =\inf \left\{ \int f \, d\mu : \mu \in {\mathcal {J}}_z^m\right\} . \end{aligned}$$

Since $\delta _z \in {\mathcal {J}}_z^m$ we have that

$$\begin{aligned} \inf \left\{ \int f \, d\mu : \mu \in {\mathcal {J}}_z^m\right\} \le \int f \, d \delta _z=f(z). \end{aligned}$$

Hence,

$$\begin{aligned} f(z)=\inf \left\{ \int u \, d\mu : \mu \in {\mathcal {J}}_z^m\right\} \qquad \text { for } z \in \partial \Omega . \end{aligned}$$

Conversely, extend *f* to a continuous function on ${\bar{\Omega }}$ (for instance one can take ${\text {PWB}}_f$, which was defined in Theorem A in the introduction) and for simplicity denote it also by *f*. Since $\Omega$ is a *m*-hyperconvex domain then by Theorem 4.1 for any $z\in \partial \Omega$ and any $\mu \in {\mathcal {J}}_z^m$ it holds that ${\text {supp}} (\mu ) \subseteq \partial \Omega$, so we have

$$\begin{aligned} f(z)=\inf \left\{ \int f \, d\mu : \mu \in {\mathcal {J}}_z^m\right\} \qquad \text { for all } z \in \partial \Omega . \end{aligned}$$

Edwards’ theorem (Theorem 2.8) gives us now that

$$\begin{aligned} \mathbf{S }^c_f(z)=\inf \left\{ \int f \, d\mu : \mu \in {\mathcal {J}}_z^m\right\} , \end{aligned}$$

and therefore $\mathbf{S }^c_f=f$ on $\partial \Omega$. To conclude this proof, we shall prove that for $z\in \partial \Omega$ it holds that

$$\begin{aligned} \lim _{\xi \rightarrow z}\mathbf{S }^c_f(\xi )=f(z). \end{aligned}$$

We shall argue by contradiction. Assume that

$$\begin{aligned} \varliminf _{\xi \rightarrow z}\mathbf{S }^c_f(\xi ) < f(z)\qquad \text { for some } z \in \partial \Omega . \end{aligned}$$

Then we can find an $\varepsilon >0$, and a sequence $\xi _j \rightarrow z$ such that

$$\begin{aligned} \mathbf{S }^c_f(\xi _j)< f(z)-\varepsilon \qquad \text { for every } j. \end{aligned}$$

Since, for every *j*, we have that

$$\begin{aligned} \mathbf{S }^c_f(\xi _j)=\inf \left\{ \int f \, d\mu : \mu \in {\mathcal {J}}_{\xi _j}^m\right\} \end{aligned}$$

there are measures $\mu _j \in {\mathcal {J}}_{\xi _j}^m$ such that

$$\begin{aligned} \int f \, d\mu _j < f(z) - \varepsilon . \end{aligned}$$

By passing to a subsequence, and using Theorem 2.9, we can assume that $\mu _j$ converges in the weak-$^*$ topology to some $\mu \in {\mathcal {J}}_z^m$. Hence,

$$\begin{aligned} \int f \, d\mu =\lim _j \int f \, d\mu _j < f(z)-\varepsilon . \end{aligned}$$

This contradicts the assumption that

$$\begin{aligned} f(z)=\inf \left\{ \int f \, d\mu : \mu \in {\mathcal {J}}_z^m\right\} \, . \end{aligned}$$

Therefore, by Corollary 2.6, $\mathbf{S }^c_f \in {\mathcal {SH}}_m(\Omega ) \cap {\mathcal {C}}({\bar{\Omega }})$, and the proof is finished. $\square$

### Remark

If $\Omega$ is a bounded domain that is not necessarily *m*-hyperconvex, then we have a similar result as in Corollary 4.2 namely that there exists a function $u \in {\mathcal {SH}}_m(\Omega ) \cap {\mathcal {C}}({\bar{\Omega }})$ such that $u=f$ on ${\bar{\Omega }}$ if, and only if, there exists a continuous extension $\varphi$ of *f* to ${\bar{\Omega }}$ such that

$$\begin{aligned} \varphi (z)=\inf \left\{ \int \varphi \, d\mu : \mu \in {\mathcal {J}}_z^m\right\} . \end{aligned}$$

We end this section by proving Theorem 4.3, and its immediate consequence. We have decided to deviate in Theorem 4.3 the notation from Definition 2.3. This to simplify the comparison with Theorem B in the introduction.

### Theorem 4.3

Assume that $\Omega$ is a bounded domain in ${\mathbb {C}}^n$, $n\ge 2$, $1\le m\le n$. Then the following assertions are equivalent.

1. $\partial \Omega$ is $B_m$-regular at every boundary point $z_0\in \partial \Omega$, in the sense that
  $$\begin{aligned} \lim _{\begin{array}{c} z\rightarrow z_0\\ z\in \Omega \end{array}} {\text {PB}}^m_f(z) =f(z_0), \end{aligned}$$
  for each continuous function $f:\partial \Omega \rightarrow {\mathbb {R}}$. Here
  $$\begin{aligned} {\text {PB}}^m_f(z)=\sup \Bigg \{v(z): v\in {\mathcal {SH}}_m(\Omega ),\; \varlimsup _{\begin{array}{c} \zeta \rightarrow \xi \\ \zeta \in \Omega \end{array}}v(\zeta )\le f(\xi ), \;\; \forall \xi \in \partial \Omega \Bigg \}. \end{aligned}$$
2. $\partial \Omega$ has a strong barrier at every point that is *m*-subharmonic;
3. $\Omega$ admits an exhaustion function $\varphi$ that is negative, smooth, *m*-subharmonic, and such that
  $$\begin{aligned} \left( \varphi (z)-|z|^2\right) \in {\mathcal {SH}}_m(\Omega )\, ; and \end{aligned}$$
4. $\partial \Omega =\partial _{{\mathcal {J}}_z^m}$ in the sense of Definition 2.7.

### Proof

$(1)\Rightarrow (2):$ Fix $z\in \partial \Omega$, and let *f* be a continuous function on $\partial \Omega$ such that $f(z)=0$ and $f(\xi )<0$ for $\xi \ne z$. Then ${\text {PB}}^m_f$ is a strong barrier at *z*.

$(2)\Rightarrow (1):$ Let $f\in {\mathcal {C}}(\partial \Omega )$. Then the upper semicontinuous regularization $({\text {PB}}^m_f)^*$ is *m*-subharmonic, and by the generalized Walsh theorem (Theorem 2.5) it is sufficient to show that

$$\begin{aligned} \lim _{\xi \rightarrow \partial \Omega }{\text {PB}}^m_f=f \end{aligned}$$

to obtain that ${\text {PB}}^m_f\in \mathcal {SH}_m(\Omega )\cap {\mathcal {C}}({\bar{\Omega }})$. Fix $w\in \partial \Omega$, and $\varepsilon >0$. Let $u_w\in \mathcal {SH}_m(\Omega )$ be a strong barrier at *w* that is *m*-subharmonic. Then there exists a constant $M>0$ such that

$$\begin{aligned} f(w)+Mu_w^*-\varepsilon \le f, \qquad \text { on }\partial \Omega , \end{aligned}$$

and therefore we have that $f(w)+Mu_w-\varepsilon \le {\text {PB}}^m_f$. This gives us that

$$\begin{aligned} \varliminf _{\xi \rightarrow w}{\text {PB}}^m_f(\xi )\ge f(w)-\varepsilon , \end{aligned}$$

and finally $$\lim _{\xi \rightarrow w}{\text {PB}}^m_f(\xi )=f(w)$$.

$(1)\Rightarrow (4):$ Fix $z\in \partial \Omega$. Let *f* be a continuous function on $\partial \Omega$ such that $f(z)=0$ and $f(\xi )<0$ for $\xi \ne z$. Then ${\text {PB}}^m_f\in \mathcal {SH}_m(\Omega )\cap {\mathcal {C}}({\bar{\Omega }})$, and ${\text {PB}}^m_f=f$ on $\partial \Omega$. Let $\mu \in {\mathcal {J}}_z^m$ then, since $\mu$ is a probability measure on ${\bar{\Omega }}$, we have that

$$\begin{aligned} {\text {PB}}^m_f(z)\le \int {\text {PB}}^m_f\, d\mu \le \left( \max _{{\text {supp}}(\mu )}{\text {PB}}^m_f\right) \int \,d\mu ={\text {PB}}^m_f(z). \end{aligned}$$

Thus, $$\mu =\delta _z$$.

$(4)\Rightarrow (1):$ This follows from Corollary 4.2.

$(1)\Rightarrow (3):$ Take $f(z)=-2|z|^2$ on $\partial \Omega$ and set $u(z)={\text {PB}}^m_f(z)+|z|^2$. By Richberg’s approximation theorem, we can find a smooth function *v* that is *m*-subharmonic and

$$\begin{aligned} \lim _{\xi \rightarrow \partial \Omega }\left( u(\xi )- v(\xi )\right) =0. \end{aligned}$$

This implication is then concluded by letting $\varphi (z)=v(z)+|z|^2$. Some comments on Richberg’s approximation theorem are in order. In our case, Demailly’s proof of Theorem 5.21 in [22] is valid. Richberg’s approximation theorem is valid in a much more abstract setting (see e.g., [33, 52]).

$(3)\Rightarrow (1):$ Let $f\in {\mathcal {C}}(\partial \Omega )$, and let $\varepsilon >0$. Then there exists a smooth function *g* defined on a neighborhood of ${\bar{\Omega }}$ such that

$$\begin{aligned} f\le g\le f+\varepsilon , \qquad \text { on } \partial \Omega . \end{aligned}$$

By assumption there exists a constant $M>0$ such that $g+M\varphi \in \mathcal {SH}_m(\Omega )$. Then we have that

$$\begin{aligned} g+M\varphi -\varepsilon \le f, \qquad \text { on } \partial \Omega . \end{aligned}$$

Hence, $g+M\varphi -\varepsilon \le {\text {PB}}^m_f$ in $\Omega$. This means that

$$\begin{aligned} \varliminf _{\xi \rightarrow w}{\text {PB}}^m_f(\xi )\ge g(w)-\varepsilon \ge f(w)-\varepsilon \qquad \text { for all } w\in \partial \Omega , \end{aligned}$$

and therefore we get

$$\begin{aligned} \lim _{\xi \rightarrow w}{\text {PB}}^m_f(\xi )=f(w). \end{aligned}$$

Thus, ${\text {PB}}^m_f\in \mathcal {SH}_m(\Omega )\cap {\mathcal {C}}({\bar{\Omega }})$, by the generalized Walsh theorem (Theorem 2.5). $\square$

An immediate consequence of Theorem 4.3 is the following corollary.

### Corollary 4.4

Let $\Omega$ be a bounded domain in ${\mathbb {C}}^n$ such that for every $z\in \partial \Omega$ there exists a neighborhood $U_z$ such that $\Omega \cap U_z$ is $B_m$-regular, then $\Omega$ is $B_m$-regular.

### Proof

Let $z\in \partial \Omega$, $U_z$ be a neighborhood of *z*, and let $u_z$ be a strong barrier at *z*, that is *m*-subharmonic, and defined in some neighborhood of ${{\bar{U}}}_z\cap \Omega$. Now let $\delta >0$, be such that $u_z<-\delta$ on $\partial U_z\cap \Omega$. Then we can define a (global) strong barrier at *z*, that is *m*-subharmonic:

$$\begin{aligned} v_z(w)= {\left\{ \begin{array}{ll} \max \{u_z(w),-\delta \} &{} \text{ if } w\in U_z\cap \Omega ,\\ -\delta &{} \text{ if } w\in \Omega \setminus U_z. \end{array}\right. } \end{aligned}$$

$$\square $$

## The Existence of Smooth Exhaustion Functions

The purpose of this section is to prove the implication $(1)\Rightarrow (3)$ in Theorem 4.1. That we shall do in Theorem 5.4. This section is based on the work of Cegrell [19], and therefore shall need a few additional preliminaries.

### Definition 5.1

Assume that $\Omega$ is a bounded domain in ${\mathbb {C}}^n$, and let $u\in \mathcal {SH}_m(\Omega )\cap L^{\infty }(\Omega )$. Then the *m-Hessian measure* of *u* is defined by

$$\begin{aligned} {\text {H}}_m(u)=(dd^cu)^m\wedge \beta ^{n-m}, \end{aligned}$$

where $$\beta =dd^c|z|^2$$.

### Remark

The *m*-Hessian measure is well-defined for much more general functions than needed in this section. For further information see e.g., [9].

For a bounded *m*-hyperconvex domain in ${\mathbb {C}}^n$, we shall use the following notation

$$\begin{aligned} {\mathcal {E}}_m^0(\Omega )=\bigg \{\varphi \in \mathcal {SH}_m(\Omega )\cap L^\infty (\Omega ): \,\varphi \le 0,\,\, \lim _{z\rightarrow \partial \Omega }\varphi (z)=0, \int _\Omega {\text {H}}_m(\varphi )<\infty \bigg \}. \end{aligned}$$

In Theorem 5.4, we shall prove that a *m*-hyperconvex domain admits an exhaustion function that is smooth, and strictly *m*-subharmonic. Our method is that of approximation. Therefore, we first need to prove a suitable approximation theorem. Theorem 5.2 was first proved in the case $m=n$ by Cegrell [19]. If the approximating sequence $\{\psi _j\}$ is assumed to be only continuous on $\Omega$, then the corresponding result was proved by Cegrell [18, Theorem 2.1] in the case $m=n$, and Lu [46, Theorem 1.7.1] for general *m*. In connection with Theorem 5.2, we would like to make a remark on Theorem 6.1 in a recent paper by Harvey et al. [34]. There they prove a similar approximation theorem, but there is an essential difference. They assume that the underlying space should admit a negative exhaustion function that is $\mathcal {C}^2$-smooth, and strictly *m*-subharmonic. Thereafter, they prove that approximation is possible. Whereas we prove that smooth approximation is always possible on an *m*-hyperconvex domain, i.e., there should only exist a negative exhaustion function. Thereafter, we prove the existence of a negative and smooth exhaustion function that is strictly *m*-subharmonic, and has bounded *m*-Hessian measure. We believe that Theorem 5.2 is of interest in its own right.

### Theorem 5.2

Assume that $\Omega$ is a bounded *m*-hyperconvex domain in ${\mathbb {C}}^n$. Then, for any negative *m*-subharmonic function *u* defined on $\Omega$, there exists a decreasing sequence $\{\psi _j\}\subset {\mathcal {E}}_m^0(\Omega )\cap {\mathcal {C}}^{\infty }(\Omega )$ such that $\psi _j\rightarrow u$, as $j\rightarrow \infty$.

Before proving Theorem 5.2, we need the following lemma. The proof is as in [19], and therefore it is omitted.

### Lemma 5.3

Let *u*, *v* be smooth *m*-subharmonic functions in $\Omega$ and let $\omega$ be a neighborhood of the set $\{u=v\}$. Then there exists a smooth *m*-subharmonic function $\varphi$ such that $\varphi \ge \max \{u,v\}$ on $\Omega$ and $\varphi =\max \{u,v\}$ on $\Omega \setminus \omega$.

Now to the proof of Theorem 5.2.

### *Proof of Theorem *5.2

By Theorem 3.5, property (3), we can always find a continuous and negative exhaustion function $\alpha$ for $\Omega$ that is strictly *m*-subharmonic.

We want to prove that for any $u\in {\mathcal {E}}_m^0(\Omega )\cap {\mathcal {C}}({\bar{\Omega }})$ with ${\text {supp}}({\text {H}}_m(u))\Subset \Omega$, and for any $a\in (1,2)$, there exists $\psi \in {\mathcal {E}}_m^0(\Omega )\cap {\mathcal {C}}^{\infty }(\Omega )$ such that

5.1 $$\begin{aligned} au\le \psi \le u. \end{aligned}$$

We shall do it in several steps.

*Step 1.* Fix a constant $s<0$ such that

$$\begin{aligned} {\text {supp}}({\text {H}}_m(u))\Subset \Omega _0=\{z \in \Omega : \alpha (z)<s\}, \end{aligned}$$

and let $1<b<a<2$ and $c<0$ be constants such that $au<bu+c$ in a neighborhood of ${\bar{\Omega }}_0$. Note that we have

$$\begin{aligned} \bar{\Omega }_0\subset \{au<c\}\subset \{2u<c\}. \end{aligned}$$

By using standard regularization by convolution (Theorem 2.2 (6)), we can construct a sequence $\phi _j'$ of smooth *m*-subharmonic functions decreasing to *bu*. Out of this sequence pick one function, $\varphi _0'$, that is smooth in a neighborhood of the set $\{2u\le c\}$, and such that $\varphi _0'<u$ on $\bar{\Omega }_0$. Next, define

$$\begin{aligned} \varphi _0= {\left\{ \begin{array}{ll} \max \{2u,\varphi _0'+c\} &{} \text { on } \{2u<c\}, \\ 2u, &{}\text { on } \{2u\ge c\}. \end{array}\right. } \end{aligned}$$

Then by construction, we have that $\varphi _0\in {\mathcal {E}}_m^0(\Omega )\cap {\mathcal {C}}({\bar{\Omega }})$. Furthermore, on a neighborhood of $\bar{\Omega }_0$ we have $\varphi _0=\varphi _0'+c$, since

$$\begin{aligned} 2u<au<bu+c<\varphi _0'+c. \end{aligned}$$

With the definition

$$\begin{aligned} {{\tilde{\varphi }}}_0=\sup \{v\in \mathcal {SH}_m(\Omega ): v\le \varphi _0 \, \text{ on } \, \Omega _0, v\le 0\}, \end{aligned}$$

we get that ${\tilde{\varphi }}_0=\mathbf{S }_f$, where

$$\begin{aligned} f= {\left\{ \begin{array}{ll} \varphi _0 &{} \text { on } \, \Omega _0, \\ h &{} \text { on } \, {\bar{\Omega }}\setminus \Omega _0, \end{array}\right. } \end{aligned}$$

is a continuous function. Here *h* is the unique harmonic function on $\Omega \setminus \Omega _0$ that is continuous up to the boundary, $h=\varphi _0$ on $\partial \Omega _0$ and $h=0$ on $\partial \Omega$. In fact the function *h* can be obtain as (see [4])

$$\begin{aligned} h(z)=\sup \left\{ \theta (z):\theta \in \mathcal {SH}_1(\Omega \setminus {\overline{\Omega }}_0), \, \limsup _{z\rightarrow \xi } \theta (z)\le \varphi _0(\zeta ),\, \forall \, \zeta \in \partial {\Omega _0}\cup \partial \Omega \right\} . \end{aligned}$$

Thanks to the generalized Walsh theorem (Theorem 2.5), we have that ${\tilde{\varphi }}_0\in \mathcal {SH}_m(\Omega )\cap {\mathcal {C}}({\bar{\Omega }})$. Furthermore,

$$\begin{aligned} au<bu+c\le \varphi _0'+c=\varphi _0={\tilde{\varphi }}_0<\varphi _0'<u\qquad \text { on }{\bar{\Omega }}_0. \end{aligned}$$

Thus, we see that

$$\begin{aligned} au<{\tilde{\varphi }}_0<u \qquad \text{ on } {\bar{\Omega }}. \end{aligned}$$

The set $\{au\le \varphi _0\}\subset \{2u\le c\}$ is compact, and therefore we have that $\varphi _0$ is smooth in a neighborhood of $\{au\le \varphi _0\}$.

*Step 2.* Let $\Omega _0'$ be a given domain such that $\Omega _0\Subset \Omega _0'\Subset \Omega$. We shall construct functions $\varphi _1$, ${\tilde{\varphi }}_1$, and a domain $\Omega _1$ with the following properties;

1. $\Omega _0'\Subset \Omega _1\Subset \Omega$ and $\Omega _1=\{\alpha <s_1\}$, for some $s_1<0$;
2. $$\varphi _1,{\tilde{\varphi }}_1\in {\mathcal {E}}_m^0(\Omega )\cap {\mathcal {C}}({\bar{\Omega }})$$;
3. $$\varphi _0=\varphi _1$$ on $$\Omega _0$$;
4. $$au<{\tilde{\varphi }}_1<u$$ on $$\Omega $$;
5. $$\varphi _1={\tilde{\varphi }}_1$$ on $$\Omega _1$$;
6. $$\{au\le \varphi _1\}\Subset \Omega $$; and
7. $\varphi _1$ is smooth in a neighborhood of $\{au\le \varphi _1\}$.

We start by taking $s_1<0$ such that

$$\begin{aligned} \Omega _0'\Subset \Omega _1=\{\alpha <s_1\}\Subset \Omega . \end{aligned}$$

and $\varphi _0<au$ on $\partial \Omega _1$. This is possible since the set $\{au\le \varphi _0\}$ is compact. Let $1<b<a$, and $c<d<0$, with the properties that

$$\begin{aligned} au<bu+d<{\tilde{\varphi }}_0 \, \text { on a neighborhood of }\, {\bar{\Omega }}_1. \end{aligned}$$

Once again using standard approximation by convolutions, let $\phi _j''$ be a sequence of smooth *m*-subharmonic functions decreasing to $bu+d$. Take one function from this sequence, call it $\varphi _1''$, such that it is smooth in a neighborhood of $\{2u\le d\}$, and

$$\begin{aligned} \varphi _1''<{\tilde{\varphi }}_0 \qquad \text { on } {\bar{\Omega }}_1. \end{aligned}$$

The definition

$$\begin{aligned} \varphi _1'= {\left\{ \begin{array}{ll} \max \{\varphi _1'',2u\} &{} \text { on } \{2u<d\}, \\ 2u &{} \text { on } \{2u\ge d\} \end{array}\right. } \end{aligned}$$

yields that $\varphi _1'\in {\mathcal {E}}_m^0(\Omega )\cap {\mathcal {C}}({\bar{\Omega }})$, and we have that $\varphi _1'=\varphi _1''$ near $\{au\le \varphi _1'\}$.

Take an open set *W* such that

$$\begin{aligned} \{au<\varphi _0=\varphi _1'\}\Subset W\Subset \{au<\min (\varphi _0,\varphi _1')\}\setminus {\bar{\Omega }}_0, \end{aligned}$$

therefore by Lemma 5.3 there exists $\varphi _1\in {\mathcal {E}}_m^0(\Omega )$ such that $\varphi _1<u$ on $\Omega$, and with $\varphi _1\ge \max \{\varphi _0,\varphi _1'\}$ with equality on $\Omega _0$. Furthermore, $\varphi _1$ is smooth on *W* and $\varphi _1=\varphi _0$ on $\Omega _0$. It also follows that $\varphi _1$ is smooth near $\{au\le \varphi _1\}$ which contains ${\bar{\Omega }}_1$, since $\varphi _1=\varphi _1'$ if $\varphi _0<au\le \varphi _1$. Both functions $\varphi _0$, and $\varphi _1'$, are smooth near

$$\begin{aligned} \{au\le \varphi _0\}\cap \{au\le \varphi _1'\}. \end{aligned}$$

Let us define

$$\begin{aligned} {\tilde{\varphi }}_1=\sup \{v\in \mathcal {SH}_m(\Omega ): v\le \varphi _1 \, \text{ on } \, \Omega _1, v\le 0\}, \end{aligned}$$

then as in *Step 1*, it follows that ${\tilde{\varphi }}_1\in \mathcal {SH}_m(\Omega )\cap {\mathcal {C}}({\bar{\Omega }})$. The constructions $\varphi _1$, ${\tilde{\varphi }}_1$ and $\Omega _1$ satisfy all the Conditions (1)–(7).

*Step 3*. Now if $\Omega _j\nearrow \Omega$, then the function

$$\begin{aligned} \psi =\lim _{j\rightarrow \infty }\varphi _j\in {\mathcal {E}}_m^0(\Omega ). \end{aligned}$$

Furthermore, $\psi$ is smooth since for any domain $\omega \Subset \Omega$ there exists $j_{\omega }$ such that on the set $\omega$ we have $\psi =\varphi _{j_{\omega }}\in {\mathcal {C}}^{\infty }$. This ends the proof of (5.1).

To finish the proof of this theorem, assume that *u* is a negative *m*-subharmonic function defined on $\Omega$. Theorem 1.7.1 in [46] implies that there exists a decreasing sequence $\{u_j\}\subset {\mathcal {E}}_m^0(\Omega )\cap {\mathcal {C}}({\bar{\Omega }})$, ${\text {supp}}({\text {H}}_m(u_j))\Subset \Omega$, such that $u_j\rightarrow u$, as $j\rightarrow \infty$. Then by (5.1), there exists a sequence $\psi _j\in {\mathcal {E}}_m^0(\Omega )\cap {\mathcal {C}}^{\infty }(\Omega )$ with

$$\begin{aligned} \left( 1-\frac{1}{j+1}\right) u_j\le \psi _j\le \left( 1-\frac{1}{j}\right) u_j, \end{aligned}$$

and the proof is finished. $\square$

We shall end this paper by proving the implication $(1)\Rightarrow (3)$ in Theorem 4.1.

### Theorem 5.4

Assume that $\Omega$ is a *m*-hyperconvex domain in ${\mathbb {C}}^n$, $n\ge 2$, $1\le m\le n$. Then $\Omega$ admits an exhaustion function that is negative, smooth, strictly *m*-subharmonic, and has bounded *m*-Hessian measure.

### Proof

Theorem 5.2 implies that there exists a function $\psi \in {\mathcal {E}}_m^0(\Omega )\cap {\mathcal {C}}^{\infty }(\Omega )$. Let $M>0$ be a constant such that

$$\begin{aligned} |z|^2-M<-1 \qquad \text { on } \Omega , \end{aligned}$$

and define

$$\begin{aligned} \psi _j(z)=\max \left\{ \psi (z),\frac{|z|^2-M}{j}\right\} \in {\mathcal {E}}_m^0(\Omega )\cap {\mathcal {C}}(\Omega ). \end{aligned}$$

This construction also implies that $\psi _j$ is smooth outside a neighborhood $\omega$ of the set

$$\begin{aligned} \left\{ \psi (z)=\frac{|z|^2-M}{j}\right\} . \end{aligned}$$

Lemma 5.3 implies that there exists $\varphi _j\in {\mathcal {E}}_m^0(\Omega )\cap {\mathcal {C}}^{\infty }(\Omega )$ such that $\varphi _j=\psi _j$ outside $\omega$. Now we choose a sequence $a_j\in (0,1)$ such that the function

$$\begin{aligned} \varphi =\sum _{j=1}^{\infty }a_j\varphi _j \end{aligned}$$

is smooth, strictly *m*-subharmonic, and belongs to ${\mathcal {E}}_m^0(\Omega )$. It is sufficient to take

$$\begin{aligned} a_j=\frac{1}{2^j\max \left\{ \Vert \varphi _j\Vert _{\infty }, h_j^{\frac{1}{m}}, 1\right\} }, \quad \text{ where } \, h_j=\int _{\Omega }{\text {H}}_m(\varphi _j). \end{aligned}$$

Note here that $|\varphi |\le 1$. The construction

$$\begin{aligned} u_n=\sum _{j=1}^na_j\varphi _j \end{aligned}$$

implies that $u_n\in {\mathcal {E}}_m^0(\Omega )$, and $u_n\searrow \varphi$, as $n\rightarrow \infty$. Using standard arguments, and finally by passing to the limit with *n*, we arrive at

$$\begin{aligned} \int _{\Omega }{\text {H}}_m(\varphi )\le \left( \sum _{j=1}^{\infty }a_j\left( \int _{\Omega }{\text {H}}_m(\varphi _j)\right) ^{\frac{1}{m}}\right) ^m\le 1. \end{aligned}$$

Let us conclude this proof by motivating why $\varphi$ is necessarily smooth, and strictly *m*-subharmonic. Let $\Omega '\Subset \Omega$, then there exists an index $j_\omega$ such that on $\Omega '$ we have that

$$\begin{aligned} \varphi _j=\frac{|z|^2-M}{j}\qquad \text { for } j>j_\omega . \end{aligned}$$

This gives us that

$$\begin{aligned} \varphi =\sum _{j=1}^{j_\omega }a_j \varphi _j+\sum _{j=j_\omega +1}^{\infty }a_j \left( \frac{|z|^2-M}{j}\right) \qquad \text{ on } \, \Omega '. \end{aligned}$$

$$\square $$
